# Synergistic Combination of Facile Thiol-Maleimide Derivatization and Supramolecular Solvent-Based Microextraction for UHPLC–HRMS Analysis of Glutathione in Biofluids

**DOI:** 10.3389/fchem.2021.786627

**Published:** 2021-12-09

**Authors:** Mengmeng Yan, Feng Gao, Meng Chen, Qi Hu, Yuqin Yang, Kedian Chen, Penglong Wang, Haimin Lei, Qiang Ma

**Affiliations:** ^1^ School of Chinese Materia Medica, Beijing University of Chinese Medicine, Beijing, China; ^2^ Chinese Academy of Inspection and Quarantine, Beijing, China; ^3^ School of Chemical Engineering, Dalian University of Technology, Dalian, China

**Keywords:** glutathione, derivatization, supramolecular solvent, dispersive liquid–liquid microextraction, quadrupole/Orbitrap high-resolution mass spectrometry, biofluid

## Abstract

Glutathione (GSH) is the most abundant non-protein thiol in biofluids, enabling diverse physiological functions. Among the proposed methods for GSH detection, ultra-high-performance liquid chromatography (UHPLC) coupled with high-resolution mass spectrometry (HRMS) has the advantages of high sensitivity and efficiency. In this study, a novel analytical method was developed for the determination of GSH using supramolecular solvent (SUPRAS)-based dispersive liquid–liquid microextraction (DLLME) and UHPLC–HRMS. N-Laurylmaleimide was dissolved in tetrahydrofuran, which served three functions: 1) precipitate the proteins present in the biofluid sample, 2) provide a reaction environment for derivatization, and 3) enable the use of SUPRAS as the dispersing agent. Critical parameters were optimized based on single factor testing and response surface methodology. The established method was validated in terms of linearity, accuracy, precision, and successful quantitative analysis of GSH in saliva, urine, and plasma samples. Experimental results showed that SUPRAS as an extraction solvent was particularly suitable for the extraction of GSH from complex matrices. The current study provides a useful tool for accurate measurements of GSH concentrations, which could potentially be used for clinical diagnostics.

## 1 Introduction

According to the World Health Organization (WHO), a biomarker refers to any substance, structure, or process that can be measured in the body or its products and influence or predict the incidence of an outcome or disease (IPCS, 2001). As an important group of biomarkers, biothiols, such as glutathione (GSH), cysteine (Cys), cysteinylglycine (CysGly), N-acetylcysteine (Nac), and homo-cysteine (Hcy) have been reported to play critical roles in a variety of pathological processes (Chen et al., 2020a). Among these biothiol biomarkers, GSH is the most abundant in cells, enabling many physiological functions (Giustarini et al., 2003; Zhu et al., 2021), including antioxidation, signal transduction, and gene regulation (Hwang et al., 1992; Hyman and Franz, 2012; Zhang et al., 2020a). Abnormal levels of GSH may induce various diseases, such as cancer, Alzheimer’s disease, and human immunodeficiency virus (HIV) (Aksenov et al., 1998; Townsend et al., 2003; Leitner et al., 2007). In light of its important biological and clinical significance, there have been increasing interest in developing analytical methods for GSH determination (Steghens et al., 2003). GSH-recycling assay (Tietze, 1969; Chowdhury et al., 2013) with commercial assay kits, fluorescence (Cai et al., 2015), electrochemistry (Gao et al., 2016), surface-enhance Raman spectroscopy (SERS) (Saha and Jana, 2013; Zhu et al., 2021), colorimetry (Lee et al., 2018), flow injection analysis (Zitka et al., 2007; Kukoc-Modun et al., 2020), and high-performance liquid chromatography–mass spectrometry (HPLC–MS) (Moore et al., 2013) have been documented. Among these methods, HPLC–MS has been a method of choice for the determination of GSH due to its advantages of sensitivity and specificity (Moral et al., 2012; Avula et al., 2013; Moore et al., 2013; Sun et al., 2019). Ultra-high-performance liquid chromatography (UHPLC) that employs columns packed with sub-two microns particles can achieve excellent separation efficiencies at optimum linear velocities (Cintrón and Colón, 2002; Discenza et al., 2010). Meanwhile, with satisfactory sensitivity in full-scan acquisition mode and high resolving power, high-resolution mass spectrometry (HRMS) is effective for both qualitative and quantitative analyses (Bijlsma et al., 2012). Thus, the combination of UHPLC and HRMS has been a powerful tool for the determination of GSH (Vallverdu-Queralt et al., 2015; Zhang et al., 2020b; Zuo et al., 2018).

Since the thiol group of GSH can be easily oxidized at room temperature, it is necessary to incorporate a proper reactant capable of preventing the thiol moiety from oxidation during chemical analysis (Giustarini et al., 2017; Chen et al., 2020a). Given its reliability, efficiency, and selectivity, thiol-maleimide chemistry allows facile reaction of thiol-containing molecules under ambient conditions (Ananda et al., 2008; Northrop et al., 2015; Oz et al., 2017). Maleimides have a double bond that can interact with GSH, yielding a reaction product with lower polarity (Healy et al., 2014) and better chromatographic retention compared to GSH. In addition to the ready oxidizability of GSH itself, the complicated biofluid matrices may pose another challenge for GSH analysis (Shahpasand-Kroner et al., 2018), resulting in compromised sensitivity and accuracy (Kondekova et al., 2014; Rajeev et al., 2019).

Over the years, a number of sample preparation methods have been developed, such as liquid–liquid extraction (LLE) (Hammad et al., 2021), solid-phase extraction (SPE) (Fedotov et al., 2019), and dispersive solid-phase extraction (DSPE) (Khashaba et al., 2021; Salehpour et al., 2021) as well as microextraction techniques including dispersive liquid–liquid microextraction (DLLME) (Rezaee et al., 2006; Zuloaga et al., 2015; Mansour and Khairy, 2017; Wu et al., 2019), solid-phase microextraction (SPME) (Hawthorne et al., 1992; Duman et al., 2020; Jagirani and Soylak, 2020), and single-drop microextraction (SDME) (Liu and Dasgupta, 1996; Choi et al., 2011; Delove Tegladza et al., 2020). DLLME is one of the most efficient methods for the separation and preconcentration of analytes due to its ease of operation and low cost (Moslemzadeh et al., 2020). However, organic solvents have been commonly used in DLLME, posing a potential threat to human health and the environment. Alternatively, supramolecular solvents (SUPRASs) as a new generation of green solvents have garnered increasing interest in many fields (Jafarvand and Shemirani, 2011; Ezoddin and Abdi, 2016; Chen et al., 2020b; Lian et al., 2020). SUPRASs are nanostructured liquids generated by self-assembly processes (Moral et al., 2012). The synthesis of SUPRASs is simple and is formed directly by dispersing amphiphiles and establishing the coacervation conditions (Rubio, 2020). As the most widely used SUPRASs, water-insoluble reverse micelles are aggregated by mixing alkyl alcohols or alkyl acids with tetrahydrofuran (THF) and water (Accioni et al., 2020; Yonny et al., 2020; Caleb and Alshana, 2021). The SUPRASs can extract analytes from aqueous samples effectively (Jinlei et al., 2021) and are particularly suitable for complex samples, such as biofluids with high extraction yields (Salatti-Dorado et al., 2017; Accioni et al., 2020; Lian et al., 2020).

In this study, SUPRAS-based DLLME and UHPLC–HRMS were used for the determination of GSH in biofluids. Different maleimide homologues were investigated as derivatizing agents to react with GSH based on thiol-maleimide chemistry. The reaction products exhibited enhanced hydrophobicity and improved efficiency for SUPRAS extraction.

## 2 Materials and Methods

### 2.1 Chemicals and Reagents

GSH with a purity of 97% was purchased from ANPEL Laboratory Technologies Inc. (Shanghai, China). An isotope-labeled standard for GSH (^13^C_2_,^15^N-GSH) was obtained from Cambridge Isotope Laboratories Inc. (Andover MA, United States). A series of maleimide analogs having purities of 98%, including N-ethylmaleimide (NEM), N-benzylmaleimide (NBM), and N-cyclohexylmaleimide (NCM) were purchased from J&K Scientific Ltd. (Beijing, China). In addition, N-laurylmaleimide (NLM) was synthesized by Shanghai Balmxy Pharmaceutic Co., Ltd. (Shanghai, China) and the purity was 97%. Artificial saliva and urine samples were purchased from Beijing Iphase Pharma Services Co., Ltd. (Beijing, China). Artificial plasma was obtained from China Resources Double-Crane Pharmaceutical Co., Ltd. (Beijng, China). Pentanol, hexanol, heptanol, octanol, nonanol, valeric acid, hexanoic acid, heptanoic acid, octanoic acid, orthodecanoic acid, and THF were purchased from Beijing InnoChem Science & Technology Co., Ltd. (Beijing, China). MS grade methanol, acetonitrile, and formic acid were purchased from Fisher Scientific (Pittsburgh, PA, United States). Ultrapure water used throughout the experiments was produced with a Millipore-Milli-Q^®^ Integral 5 water purification system (Bedford, MA, United States). Stock standard solutions of GSH and ^13^C_2_,^15^N-GSH were prepared at a concentration of 1.0 mg/ml in ultrapure water, from which the working standard solutions were created with water. The maleimide analogs were dissolved with THF at a concentration of 1 M as the stock standard solutions and the working standard solution concentration for this study was further diluted with THF.

### 2.2 Sample Preparation

**FIGURE 1:**
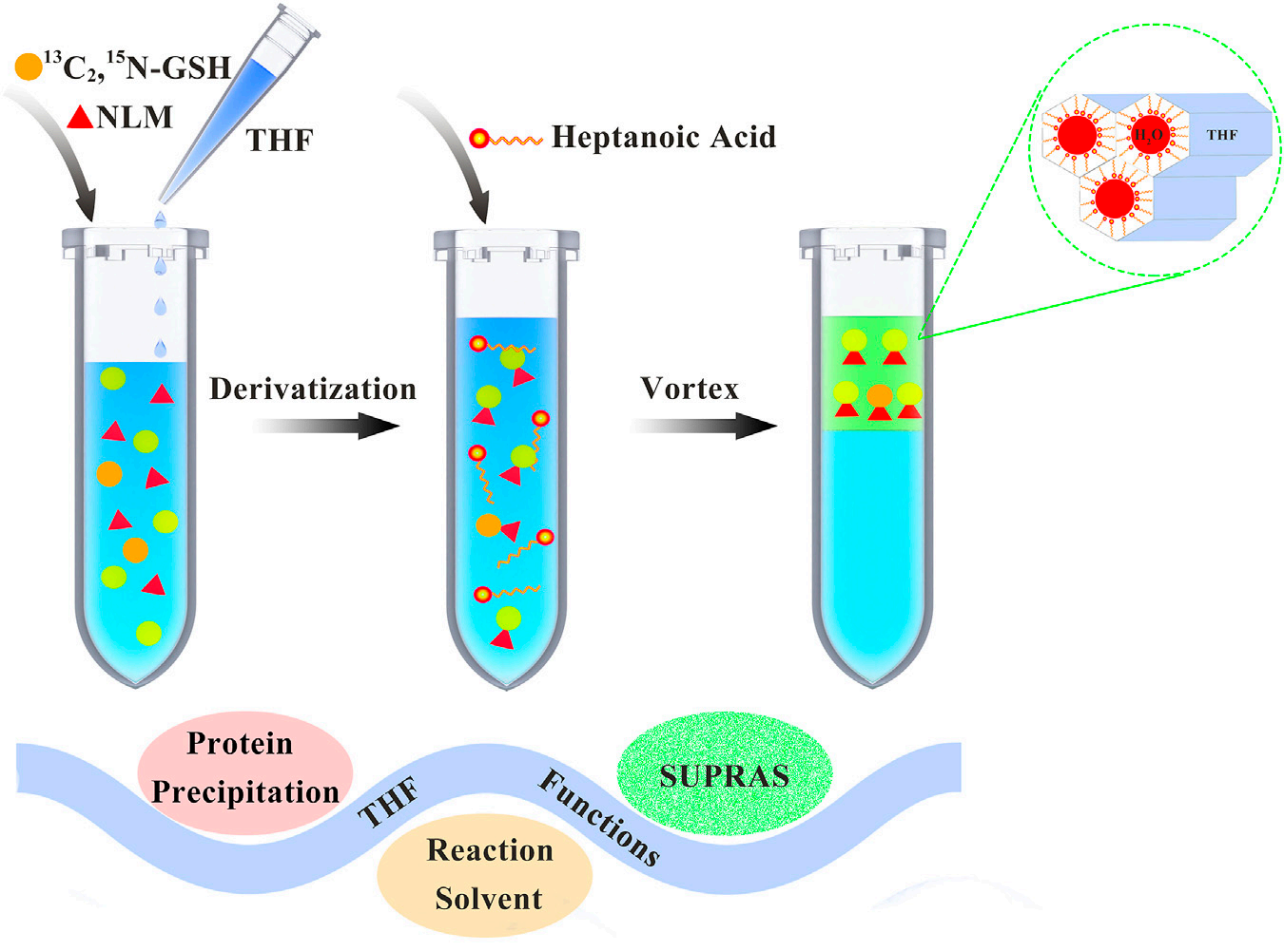
Schematic diagram of thiol-maleimide derivatization and supramolecular solvent-based microextraction.

The process of sample preparation is shown in Figure 1. Aliquots of 1 ml biofluid samples were mixed with 10 μl of the isotope internal standard (5 μg/ml), 0.66 ml THF, and 10 μl of NLM at a final concentration of 10 mM. After a vortex-assisted thiol-maleimide derivatization reaction for about 1 h, 0.23 ml of heptanoic acid was added. The mixture was vortexed again for 2 min on a vortex apparatus (Kylin-Bell Lab Instruments Co., Ltd., Haimen, China), followed by centrifugation at 3,000 rpm for 1 min using a CR 21 N high-speed refrigerated centrifuge (Hitachi, Tokyo, Japan). A 0.2 ml quantity of the resulting SUPRAS supernatant containing the analyte was collected and diluted 1:1 (v/v) with methanol for instrumental analysis.

### 2.3 UHPLC–HRMS Analysis

UHPLC separation was performed using an UltiMate 3000 liquid chromatograph equipped with a quaternary solvent pump, an online degasser, a PAL autosampler (CTC Analytics, Zwingen, Switzerland), and a thermostatted column compartment (Thermo Scientific, Sunnyvale, CA, United States). Chromatographic separation was conducted on a Waters ACQUITY UPLC HSS T3 analytical column (2.1 mm × 100 mm, 1.8 μm) at a flow rate of 0.3 ml/min. The column temperature was maintained at 30°C. A sampling volume of 2 μl was injected for each analysis. The mobile phase consisted of 0.1% (v/v) formic acid solution as the aqueous phase (A) and 0.1% (v/v) formic acid in methanol as the organic phase (B) using a gradient elution program. Initial gradient conditions were set at 10% B and held for 0.5 min. From 0.5 to 3 min, the gradient was increased incrementally to 90% B and then held at 90% B for 3 min. Finally, the gradient was returned to the initial conditions at 10 min, which completed an entire run. HRMS analysis was performed on a benchtop Q Exactive hybrid quadrupole-Orbitrap mass spectrometer (Thermo Scientific, Bremen, Germany) coupled with an electrospray ionization (ESI) source operated in positive ion mode. The analysis was performed by the full-scan MS/data-dependent MS/MS (full-scan MS^1^/dd-MS^2^) acquisition mode. Source parameters were set as follows: spray voltage, 3.8 kV; sheath gas pressure, 40 arb; auxiliary gas pressure, 10 arb; sweep gas pressure, 0 arb; capillary temperature, 350°C; and auxiliary gas heater temperature, 320°C. The analyzer scanned within the range of *m/z* 100–650 at a resolution of 70,000 full width at half maximum (FWHM) in the full MS scan mode. Automatic gain control (AGC) target value was set at 1 × 10^6^ with a maximum injection time (IT) of 100 ms. The dd-MS^2^ confirmation mode was conducted at a mass resolution of 17,500 FWHM using an isolation window of 3.0 m*/z*. Three collision energy steps were applied at 15, 25, and 35 eV. Full-scan data was used for quantitative analysis and dd-MS^2^ confirmation mode was used for confirmatory analysis. The details of UHPLC–HRMS conditions are listed in Table 1. Data acquisition and analysis were achieved using Xcalibur version 2.3 and TraceFinder version 4.1 software (Thermo Scientific, Bremen, Germany).

**TABLE 1:** Information on the retention time and precursor ions of GSH-NLM and ^13^C_2_,^15^N-GSH-NLM.

Analyte	Retention time (min)	Precursor ion (*m/z*, Δppm)			
		Identity	Theoretical	Experimental	Mass accuracy
GSH-NLM	4.75	[M + H]^+^	573.2953	573.2962	1.57
^13^C_2_,^15^N-GSH-NLM	4.75	[M + H]^+^	576.2990	576.2981	−1.56

### 2.4 Synthesis of *S-N-Laurylmaleimide glutathione* (GSH-NLM)

In this method, NLM, as the derivatization reagent, reacted with GSH to get GSH-NLM. The structure of GSH-NLM, which was subsequently extracted by SUPRAS, is shown in Supplementary Figure S1. Based on the SciFinder Scholar database, there is no report on the product of GSH-NLM. To ensure the accuracy of the experiment, the monomer of GSH-NLM was synthesized based on relevant literature, as shown in Supplementary Scheme S1. According to a previous report (Healy et al., 2014), the coupling reactions between GSH and NLM were performed simply using 90% (v/v) aqueous methanol solution. GSH and NLM were respectively dissolved in a small amount of water and THF. Then, an appropriate amount of 90% (v/v) aqueous methanol solution was added. The reaction was stirred at room temperature for about 1 h. As a result, a white precipitate formed from the reaction solution, which was washed twice with water, THF, methanol, and dichloromethane. Finally, a white powder was obtained after drying overnight in an oven set to 50°C (Yamato, Tokyo, Japan). The structure of GSH-NLM was characterized using the Q Exactive hybrid quadrupole-Orbitrap mass spectrometer, a Bruker ADVANCE III HD 400 MHz NMR spectrometer (Rheinstetten, Germany), and an X-5 micro melting point apparatus (Beijing Tech Instrument Co., Ltd., Beijing, China).

## 3 Results

### 3.1 Selection of Derivatization Reagents

**FIGURE 2:**
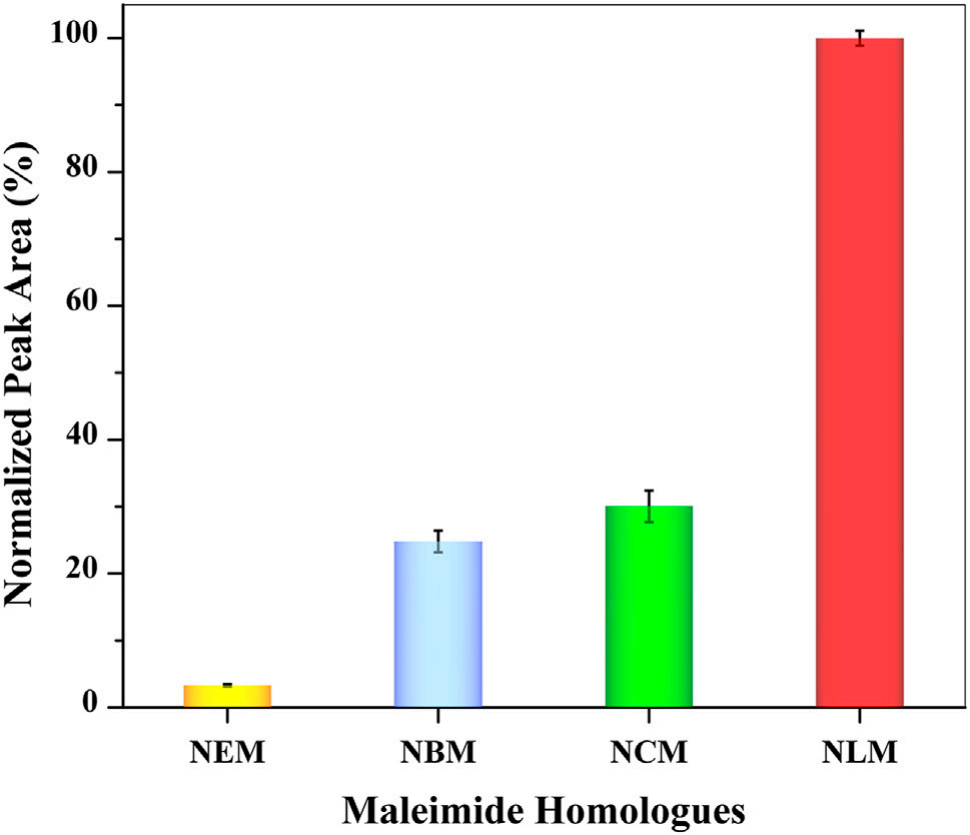
Effect of maleimide homologues types on extraction efficiency (*n* = 3).

There are various maleimide homologues that can react with GSH based on thiol-maleimide chemistry. In this study, to select the best derivatization reagent for an optimum extraction efficiency, four maleimide homologues (NEM**,** NBM, NCM, and NLM) were chosen. Detailed information on the four maleimide homologues and the structure and octanol/water partition coefficients of derivative products are listed in Supplementary Table S1. The same amount of the four derivatization reagents were each dissolved in THF. The type of derivatization reagent was the only variable. After reacting with GSH, the mixture was added to valeric acid to form SUPRAS for extraction. The upper SUPRAS layer was analyzed by UHPLC–HRMS after diluted by methanol. The relative extraction efficiencies of the four derivatization reagents are shown in Figure 2, which shows that NLM appeared to have the best extraction efficiency.

### 3.2 Characterization of GSH-NLM

GSH-NLM: white solid; melting point, 206.9°C. ^1^H-NMR (400 MHz, DMSO-*d*_*6*_) δ (ppm): 8.64 (s, 1H, -NH), 8.50 (d, *J* = 8.0 Hz, 1H, -NH), 4.48 (m, 1H, -CH), 4.01 (m, 1H, -CH), 3.69 (d, 2H, -CH_2_), 2.60–3.40 (7H, methylene- and methylidyne-), 2.34 (m, 2H, -CH_2_), 1.91 (m, 2H, -CH_2_), 1.44 (m, 2H, -CH_2_), 1.23 (s, 18H, -CH_2_), and 0.85 (t, *J* = 6.8 Hz, 5.6 Hz, 3H, -CH_3_). ^13^C-NMR (100 MHz, DMSO-*d*_*6*_) δ (ppm): 176.73, 175.08, 171.86, 170.95, 170.48, 170.34, 53.06, 52.39, 52.01, 41.27, 38.14, 35.83, 32.82, 31.42, 31.30, 29.03, 29.01, 28.97, 28.88, 28.71, 28.53, 26.98, 26.78, 26.12, 22.10, and 13.96. The ^1^H-NMR and ^13^C-NMR spectra of GSH-NLM are shown in Supplementary Figure S2. The HRMS product ion spectrum of protonated GSH-NLM is shown in Supplementary Figure S3.

### 3.3 Optimization of UHPLC–HRMS Conditions

A series of parameters, including stationary and mobile phases, flow rate, and column temperature were investigated in order to obtain optimal chromatographic separation and analytical sensitivity. Various UHPLC columns with sub-2 μm particles were compared (e.g., BEH C_18_, BEH HILIC, BEH C_8_, and HSS T3), all with the dimension of 100 mm × 2.1 mm, and 1.7 μm or 1.8 μm in particle size. The columns were paired with different mobile phase compositions consisting of acetonitrile/methanol and water to acquire satisfactory chromatographic performance by varying gradient elution programs. The results revealed that the HSS T3 column together with a methanol‒water mobile phase can achieve the best performance. Considering there are a few alkaline functional groups in the chemical structure of GSH-NLM and that it exhibits better mass spectrometric responses in positive ionization mode, the addition of 0.1% (v/v) formic acid into the aqueous and organic phases was found to be beneficial for both peak shape and signal intensity. In addition, the influences of column temperature and flow rate were studied within the ranges of 20°C–50°C and 0.2–0.5 ml/min, respectively. Ultimately, a column temperature of 30°C and a flow rate of 0.3 ml/min resulted in optimum performance.

The mass spectrometric parameters that affect the ion response of the analyte were thoroughly adjusted, including spray voltage, capillary temperature, auxiliary gas heater temperature, and flow rates of sheath gas, auxiliary gas, and sweep gas. The UHPLC–HRMS measurement was run by the full-scan MS^1^/dd-MS^2^ acquisition mode, where the precursor ion of the target analyte was first evaluated using the full MS scan event in the range of *m/z* 100–650 at a mass resolution of 70,000 FWHM, followed by a dd-MS^2^ confirmation mode with a mass resolution of 17,500 FWHM. Diagnostic product ions can be extracted using a narrow mass tolerance window (±5 ppm in this study) to reduce chemical noise.

### 3.4 Optimization of SUPRAS-Based DLLME

In the proposed sample preparation method, there are several factors that affect the pretreatment efficiency, including the type and amount of amphiphile, vortex time, centrifugation speed and time, as well as amount of THF which was used not only in the derivatization process but also in the formation of SUPRAS. In this experiment, 1.0 ml of ultrapure water containing 0.5 μg/ml GSH was used under sample preparation conditions.

#### 3.4.1 Optimization of SUPRAS-Based DLLME by Single Factor Testing

##### 3.4.1.1 Effect of THF Amount

**FIGURE 3:**
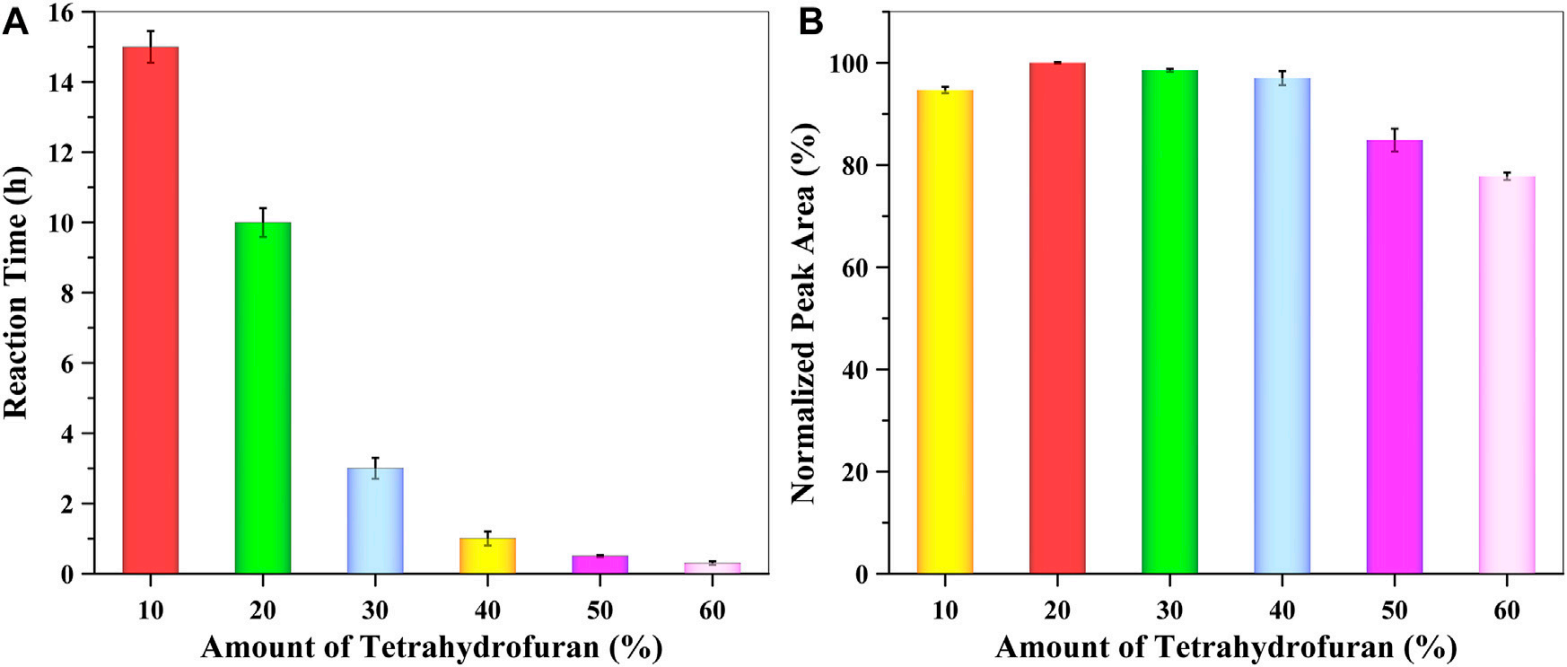
Effect of the amount of THF on **(A)** reaction time and **(B)** extraction efficiency (*n* = 3).

THF serves three functions in this experiment, two of them being optimization factors. One of its functions is to dissolve NLM. In addition, the ratio of THF as the reactor solvent can have an effect on the speed of reaction. Thus, the reaction of different amounts of THF from 10 to 60% was evaluated. Based on the complete reaction of the spiked GSH, as shown in Figure 3A, the derivatization reaction accelerated as the ratio of THF increased. When the ratio of THF was higher than 40%, the time to complete the GSH reaction was less than 1 h. On the other hand, THF may cause self-assembly of the extraction solvent, which is composed of reverse micelles. To evaluate the effect of THF amount on extraction efficiency, a series of mixture solutions were prepared using different amounts of THF, which was similar to the above procedure and under the same experimental conditions. The obtained data (Figure 3B) showed that the extraction efficiency decreased as the ratio of THF increased. Considering both the reaction time and extraction efficiency, 40% THF was selected as an optimum condition for subsequent experiments.

##### 3.4.1.2 Effect of Type and Amount of Amphiphile

**FIGURE 4:**
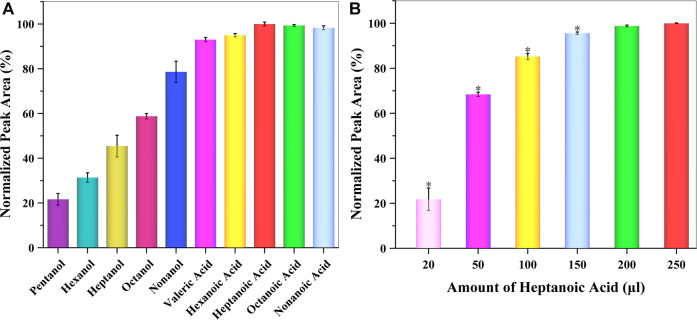
Effect of the **(A)** type and **(B)** amount of amphiphile on extraction efficiency (*n* = 3; *p < 0.05).

Besides THF, one of the most initial components of reverse micelles was amphiphile, which consisted of alkyl alcohols or alkyl acids. For appropriate extraction and preconcentration of GSH-NLM with a suitable cloudy solution, different types of amphiphiles were studied, including pentanol, hexanol, heptanol, octanol, nonanol, valeric acid, hexanoic acid, heptanoic acid, octanoic acid, and nonanoic acid. The obtained data showed that whether alkyl alcohols or alkyl acids were present, the extraction efficiency was better as the polarity of the amphoteric solvent decreased. However, the extraction efficiency of alkyl acids was better than alkyl alcohols. Among those tested, heptanoic acid was chosen as the best amphiphile in the SUPRAS formation for subsequent experiments (Figure 4A). The amount of heptanoic acid greatly influenced both the volume of SUPRAS and the resulting extraction efficiency. For this reason, the amount of heptanoic acid was evaluated in the range of 20–250 μl. The obtained data is summarized in Figure 4B, which shows that the extraction efficiency increased as the amount of heptanoic acid increased. Taking into consideration both extraction and cost efficiency, 0.2 ml of heptanoic acid was used as the optimum extraction solvent volume to extract GSH-NLM into the SUPRAS.

##### 3.4.1.3 Effect of Vortex Time

**FIGURE 5:**
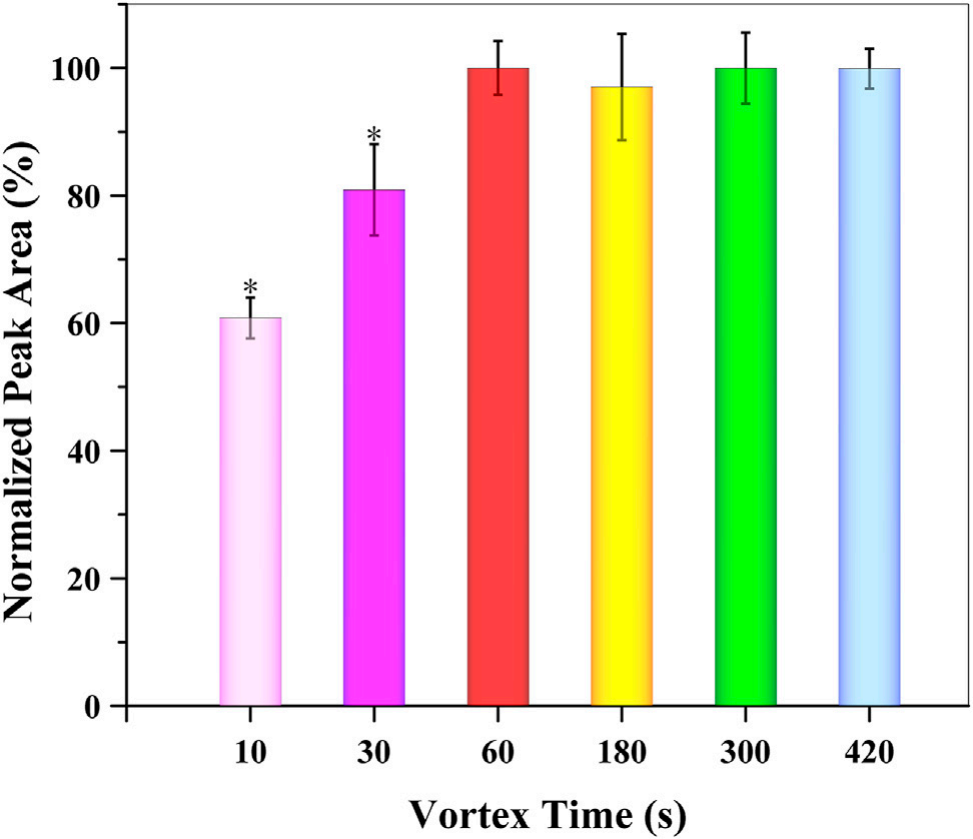
Effect of vortex time on extraction efficiency (*n* = 3; *p < 0.05).

In the SUPRAS-based DLLME procedure, a vortex-assisted process was used to not only facilitate the formation of SUPRAS but also increase the contact between the SUPRAS and target analyte. The effect of vortex time on the extraction efficiency was evaluated between 10 and 420 s. As shown in Figure 5, the extraction efficiency reached its maximum at 60 s. The best extraction efficiency was obtained after a vortex time of 60 s (1 min), which was set as the most appropriate time for the extraction of GSH-NLM by the SUPRAS.

##### 3.4.1.4 Effect of Centrifugation Time and Speed

**FIGURE 6:**
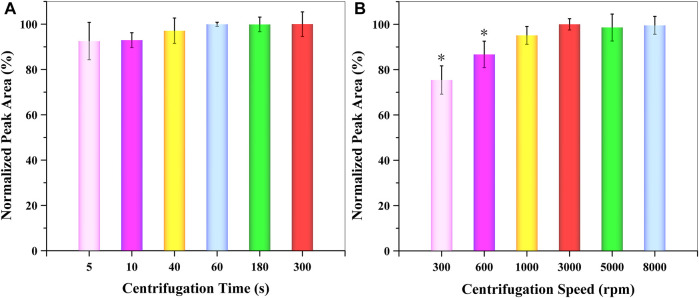
Effect of **(A)** centrifugation time and **(B)** speed on extraction efficiency (*n* = 3; *p < 0.05).

For the separation of the extraction organic phase from the aqueous solution, the centrifugation time and speed needed be evaluated and optimized. For this reason, the influence of centrifugation time and speed were examined between 5 and 300 s and 300–8,000 rpm, respectively. According to the obtained data (Figure 6), centrifugation speed had a larger effect than centrifugation time. The extraction efficiency of GSH-NLM reached a maximum at 60 s and 3,000 rpm, respectively. Thus, a centrifugation time of 60 s (1 min) and speed of 3,000 rpm were applied for the separation of the organic extraction solvent from an aqueous solution.

#### 3.4.2 Optimization of SUPRAS-Based DLLME by Response Surface Methodology

The design of experiments is used to reasonably establish conditions for optimizing the maximum extraction efficiency of GSH-NLM from aqueous samples. Based on the obtained data of single factor testing (Figures 4–6), the factors with significant differences under the set experimental conditions (*p* < 0.05) were chosen for the next experiment. Therefore, a comprehensive analysis of three key variables (amount of heptanoic acid, vortex time, and centrifugation speed) was conducted to determine their influences on extraction efficiency. The interactions between the selected variables were analyzed using the response surface methodology based on a three factor-three level Box-Behnken central composite design. The results of experimental data processing with coded levels are shown in Supplementary Tables S2, S3. All experiments were conducted in triplicate. The extraction efficiency was evaluated using the following equation:

Extraction Efficiency (%) = *m* (GSH-NLM_SUPRAS_)/(*m* (GSH-NLM_Water_) + *m* (GSH-NLM_SUPRAS_)) × 100.

**FIGURE 7:**
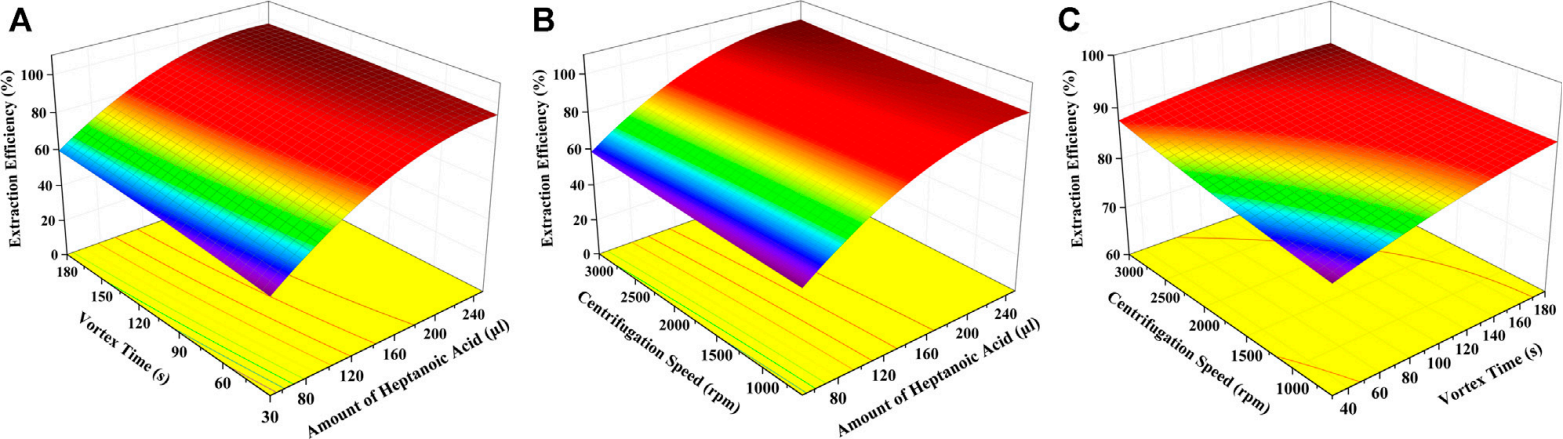
Response surface plots of different variable combinations: **(A)** amount of heptanoic acid and vortex time, **(B)** amount of heptanoic acid and centrifugation speed, and **(C)** vortex time and centrifugation speed.

The results obtained were submitted to analysis of variance (ANOVA) on Design-Expert version 8.0.6 software, and the resulting regression model was represented by the following equation:

*Y* = 14.31887 + 0.58475*X1* + 0.14121*X2* + 2.79810E-3*X3* − 2.30957E-4*X1X2* − 2.50398E-6*X1X3* − 1.50660E-5*X2X3* − 1.16272E-3*X1^2^* − 1.73614E-4*X2^2^* + 3.02513E-7*X3^2^*.

where *X1*, *X2,* and *X3* represent the amount of heptanoic acid (μl), vortex time (s), and centrifugation speed (rpm), respectively, and *Y* is the extraction efficiency (%) of GSH-NLM. ANOVA results (Table 2) of the quadratic regression model showed a *p*-value < 0.0001, demonstrating that the fitting model is a highly significant model. The model can sufficiently describe data variation while representing the actual relationship between the three variables, as evidenced by the *p*-value of lack of fit value of 0.7045. The coefficient of determination (*R* ^2^) was calculated to be 0.9973, which indicated a 99.73% variability of the response variable. The calculated coefficient of variation was 1.54%, which clearly indicated a high degree of precision and good reliability of the experimental data. The results also showed that the linear effect (*X1*, *X2,* and *X3*), quadratic effect (*X1* ^2^), and cross-product effect (*X1X2*) of the fitting model were significant, with *p* values less than 0.05. The results indicated that the amount of heptanoic acid, vortex time, and centrifugation speed, and interaction between the amount of heptanoic acid and vortex time were significantly correlated with the extraction efficiency of GSH-NLM. Other interactions between the other coefficients were not significant. The response surface plots of different variable combinations are shown in Figure 7, which can directly reflect the interactive effects of the three factors on the extraction efficiency. The optimal extraction conditions were as follows: 0.23 ml of heptanoic acid, 120 s of vortex time, and 3,000 rpm of centrifugation speed. Under these conditions, the model predicted that the extraction efficiency of GSH-NLM was 99.14%. The experimental extraction efficiency reached 98.62% with a relative error of −0.5% compared with the predicted value.

**TABLE 2:** ANOVA results for the response surface quadratic model.

Source	Sum of squares	Degrees of freedom	Mean square	*F*-value	*p*-value	
Model	4,159.68	9	462.19	289.78	<0.0001	Significant
*X1*	3,433.71	1	3,433.71	2,152.83	<0.0001	
*X2*	83.17	1	83.17	52.15	0.0002	
*X3*	42.89	1	42.89	26.89	0.0013	
*X1X2*	12.00	1	12.00	7.52	0.0288	
*X1X3*	0.36	1	0.36	0.23	0.6487	
*X2X3*	7.35	1	7.35	4.61	0.0689	
*X1* ^*2*^	569.23	1	569.23	356.89	<0.0001	
*X2* ^*2*^	4.02	1	4.02	2.52	0.1566	
*X3* ^*2*^	0.80	1	0.80	0.50	0.5020	
Residual	11.16	7	1.59			
Lack of fit	3.03	3	1.01	0.50	0.7045	Not significant
Pure error	8.14	4	2.03			
Cor total	4,170.84	16				

### 3.5 Method Validation

The established method was validated in terms of linearity, accuracy, precision, and successful quantitative analysis of GSH in saliva, urine, and plasma. A calibration curve was plotted using the matrix-matched standard solutions within the concentration range of 0.01–1 μg/ml. Good linearity was achieved by linear regression of the peak area ratio of GSH-NLM against ^13^C_2_,^15^N-GSH-NLM (y) versus the concentration of GSH (x), with the correlation coefficient (*r*) of 0.9999. The limit of detection (LOD) and quantitation (LOQ) based on signal-to-noise ratio (S/N) of 3 and 10 were 5 and 10 μg/l, respectively. The intra-day and inter-day precisions evaluated at three concentrations (10, 50, and 500 μg/l) were in the range of 1.11–6.03% and 1.26–5.97%, respectively (Supplementary Table S4). As Supplementary Table S5 shows, the average recoveries (*n* = 3) in the three biofluids at three spiked levels were 95.22–104.75% with relative standard deviations (RSDs) of 1.15–8.17%.

## 4 Discussion

GSH is an important biomarker in organisms and it is advantageous to understand its presence in the body. However, due to the particularity of its structure, it oxidizes easily in external environments, resulting in inaccurate detection. Therefore, it is very important to establish an accurate and stable detection method of GSH. However, there are some shortcomings in the current analytical methods for GSH. For example, GSH-recycling assay, the most popular method, involves the oxidation of GSH by the sulfhydryl reagent 5,5′-dithio-bis(2-nitrobenzoic acid) (DTNB) to form the yellow derivative 5′-thio-2-nitrobenzoic acid (TNB), measurable at 412 nm by a UV detector. The key to this method is glutathione reductase, which can restore oxidized glutathione (GSSG) to GSH. This makes the method very specific to GSH detection. However, the detection of GSH using GSH-recycling assay is indirect so that the total content of GSH is subtracted from the content of GSSG. Moreover, this method has strict requirements on the experimental environment and personnel. In addition, fluorescence analysis based on chemical derivatization or fluorescent probe lacks specificity because of the complexity of biofluids that contain substances similar to GSH. Meanwhile, other methods including electrochemical analysis, SERS and colorimetry have also been used for GSH detection. Although these strategies show promising results for GSH detection, without separation, matrix components present in complex biofluids may cause interferences inevitably, resulting in inaccurate results and low sensitivity. Therefore, in this study, a UHPLC–HRMS methodology with high sensitivity, good specificity, and high separation efficiency was used to detect GSH. Comparison between the current method and those reported in the literature is shown in Supplementary Table S6.

Since GSH is widely present in biological samples, a large volume of organic solvent is necessary to precipitate the protein and dilute the concentration of GSH before it can be analyzed by UHPLC–HRMS. To achieve this, SUPRASs were utilized in this study. SUPRASs are a new green extraction solvent that are widely used in various fields. The SUPRAS-based sample pretreatment method has the advantages of high extraction efficiency, low matrix effects, and enrichment that is suitable for the preparation of biological samples containing complex substances.

Based on thiol-maleimide chemistry, GSH was reacted with NLM, a maleimide homologue. This derivatization method prevents GSH from oxidizing and also reduces the polarity of GSH so that it can be separated from other polar compounds in the matrix. In this study, NLM was selected as the derivatization reagent through comparison. The derivative product of NLM and GSH has never been reported. For the accuracy of this study, GSH-NLM was synthesized, and its structure was confirmed. The compound has both a polar component including amidogen and carboxyl, and a nonpolar carbon chain, so it can only be dissolved by dimethyl sulphoxide.

During sample pretreatment, NLM was dissolved in THF and then added to the sample. Moreover, THF serves three purposes in this study: protein precipitation, reaction environment for derivatization, and formation of SUPRASs. For biological samples, organic solvents need to be added to precipitate proteins before analysis to reduce interference. Based on the comparison of mass spectra within the range of *m/z* 100–650 with and without THF added to artificial saliva, urine, and plasma (Supplementary Figure S4), THF can effectively precipitate the protein and reduce the matrix effect. According to the previous reports, due to the three-dimensional (THF-amphiphile-water) aggregates containing regions of different polarity, SUPRASs can be used as an effective medium for extraction by multiple interaction forces with organic substances including hydrogen bond, ionic, dipole–dipole, and hydrophobic interactions. Thus, the amphiphile was also optimized. According to the obtained data, compared with alkyl alcohols, alkyl acids have higher extraction efficiency to extract GSH-NLM. It can be inferred from this that hydrogen bond, ionic, and Van der Waals interactions may be the main interaction forces for the extraction (Supplementary Figure S5). Because of the amino group, these interaction forces, especially ionic interaction, played an important role.

In order to study the applicability of the method to biological samples, the application of the method in artificial plasma, saliva, and urine was explored. As a result, the recovery rates were good in the three sample matrices. This method is applicable to complex biological samples and can potentially be used to study compounds in biological samples.

## 5 Conclusion

In this study, SUPRAS-based DLLME and UHPLC–HRMS were used for the determination of GSH. In order to obtain better extraction efficiency, the types of GSH derivatization reagents (maleimides) and amphoteric substances in SUPRASs were both optimized. In addition, parameters that affect extraction efficiency such as the amounts of the amphiphilic compounds and THF, vortex time, and centrifugation speed and time were investigated. Under the optimal conditions, the established method was validated in terms of linearity, accuracy, precision, and its ability to quantitatively analyze GSH in saliva, urine, and plasma samples. Experimental results showed that SUPRAS as an extraction solvent was particularly suitable for the extraction of GSH from complex matrices. The current study provides a useful tool for accurate measurements of GSH concentrations, which could potentially be used for clinical diagnostics.

## Data Availability

The original contributions presented in the study are included in the article/Supplementary Material. Further inquiries can be directed to the corresponding authors.
