# Supplementary material for: Synergistic Combination of Facile Thiol-Maleimide Derivatization and Supramolecular Solvent-Based Microextraction for UHPLC–HRMS Analysis of Glutathione in Biofluids
Source: Front Chem. 2021 Dec 9;9:786627. doi: 10.3389/fchem.2021.786627 (PMC8695729; doi:10.3389/fchem.2021.786627)
Supplement: Supplementary file 1 [file Presentation1.pdf]

Supplementary Material

Synergistic Combination of Facile Thiol-Maleimide Derivatization and Supramolecular Solvent-Based Microextraction for UHPLC–HRMS Analysis of Glutathione in Biofluids

Mengmeng Yan[1,2], Feng Gao[1], Meng Chen[2], Qi Hu[2,3], Yuqin Yang[1], Kedian Chen[1], Penglong Wang[1], Haimin Lei[1*], and Qiang Ma[2*]

[1] School of Chinese Materia Medica, Beijing University of Chinese Medicine, Beijing 102488, China
[2] Chinese Academy of Inspection and Quarantine, Beijing 100176, China
[3] School of Chemical Engineering, Dalian University of Technology, Dalian 116024, China

[*]Correspondence authors:

Prof. Haimin Lei
School of Chinese Materia Medica, Beijing University of Chinese Medicine
Beijing 102488, China
Email: leihaimin@126.com
Phone: +86 10 8473

Prof. Qiang Ma
Chinese Academy of Inspection and Quarantine
Beijing 100176, China
Email: maqiang@caiq.org.cn
Phone: +86 10 53897463

Table of Contents

Supplementary Scheme:

Scheme S1: Derivatization reaction between glutathione (GSH) and N-laurylmaleimide (NLM).

Supplementary Tables:

Table S1: Structures and octanol/water partition coefficients (logK_{ow}) of the derivative products of the four maleimide homologues with GSH.

Table S2: Box-Behnken central composite design factors and code levels.

Table S3: Results of the Box-Behnken central composite design.

Table S4: LOD, LOQ, intra- and inter-day precision for GSH-NLM.

Table S5: Recoveries of GSH in artificial biofluids (n = 3).

Table S6: Comparison between the current method and those reported in the literature.

Supplementary Figures:

Figure S1: Structure of GSH-NLM.

Figure S2: (A) ^{1}H-NMR and (B) ^{13}C-NMR spectra of GSH-NLM.

Figure S3: Product ion spectrum of protonated GSH-NLM at *m/z* 573.2962.

Figure S4: Mass spectra of artificial biofluids spiked with GSH-NLM, exhibited within the range of *m/z* 500–600: saliva treated (A) without and (a) with THF; urine treated (B) without and (b) with THF; plasma treated (C) without and (c) with THF.

Figure S5: Proposed interactions between GSH-NLM and SUPRAS during the DLLME process.

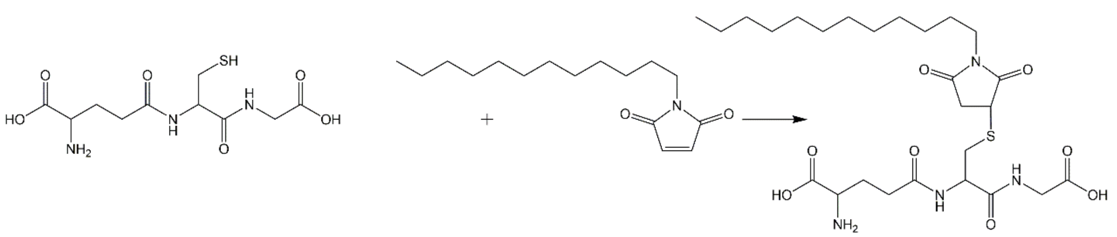

GSH NLM GSH-NLM

Scheme S1. Derivatization reaction between glutathione (GSH) and N-laurylmaleimide (NLM).

Table S1. Structures and octanol/water partition coefficients (logK_{ow}) of the derivative products of the four maleimide homologues with GSH.

Maleimide homologue	CAS	Structure	logK_{ow}
N-Ethylmaleimide (NEM)	128-53-0	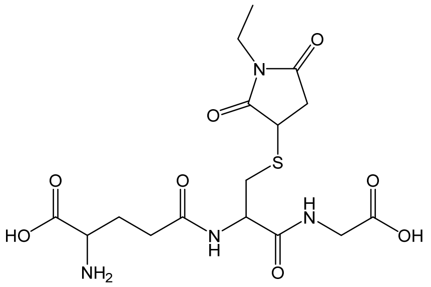	–5.67
N-Benzylmaleimide (NBM)	1631-26-1	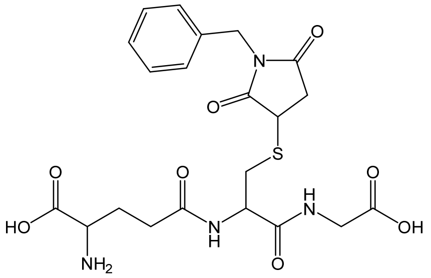	–4.45
N-Cyclohexylmaleimide (NCM)	1631-25-0	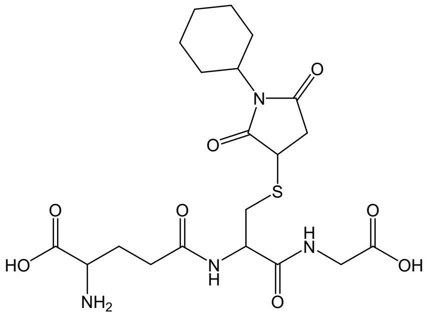	–3.89
N-Laurylmaleimide (NLM)	17616-03-4	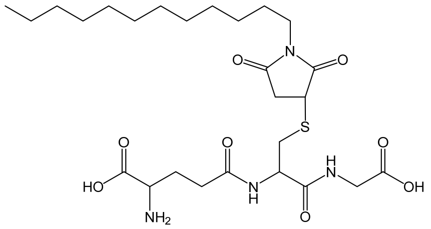	–0.76

Table S2. Box-Behnken central composite design factors and code levels.

Independent variable	Unit	Symbol	Range and level		
			–1	0	1
Amount of heptanoic acid	µl	*X1*	50	150	250
Vortex time	s	*X2*	30	105	180
Centrifugation speed	rpm	*X3*	600	1800	3000

Table S3. Results of the Box-Behnken central composite design.

No.	*X1*: Amount of heptanoic acid	*X2*: Vortex time	*X3*: Centrifugation speed	*Y:* Extraction yield of GSH-NLM (%)
1	–1	–1	0	48.8404
2	1	–1	0	93.6171
3	–1	1	0	59.9174
4	1	1	0	97.7654
5	–1	0	–1	53.2421
6	1	0	–1	95.4006
7	–1	0	1	58.0949
8	1	0	1	99.0515
9	0	–1	–1	80.5944
10	0	1	–1	88.5912
11	0	–1	1	88.3166
12	0	1	1	90.8896
13	0	0	0	87.7346
14	0	0	0	88.6737
15	0	0	0	87.4071
16	0	0	0	85.3729
17	0	0	0	89.0062

Table S4. LOD, LOQ, intra- and inter-day precision for GSH-NLM.

LOD (μg/l)	LOQ (μg/l)	Intra-day precision (%, *n* = 3) (μg/l)			Inter-day precision (%, *n* = 3) (μg/l)		
		10	50	500	10	50	500
5	10	3.75	6.03	1.11	5.97	3.21	1.26

Table S5. Recoveries of GSH in artificial biofluids (n = 3).

Concentration (μg/l)	Saliva		Urine		Plasma	
	Recovery (%)	RSD (%)	Recovery (%)	RSD (%)	Recovery (%)	RSD (%)
10	99.15	4.56	95.22	1.15	104.75	4.36
50	103.04	1.26	96.04	8.17	100.46	3.63
500	97.76	4.17	97.19	6.35	103.24	5.94

Table S6. Comparison between the current method and those reported in the literature.

Method	LOD	LOQ	Range	Sample	Reference
Current method	5 µg/l	10 µg/l	0.01–1 µg/ml	Saliva/Urine/Plasma	Our work
GSH-recycling assay	–	0.103 µM	0.103–26.4 µM	Blood/Tissues	Rahman et al., 2006
Fluorescence	300 nM	1 µM	1–10 µM	Human serum	Cai et al., 2015
Electrochemistry	–	5 µM	5–875 µM	Eye drops	Yuan et al., 2013
Surface-enhance Raman spectroscopy	10 nM	50 nM	50–1500 nM	Cells	Zhu et al., 2020

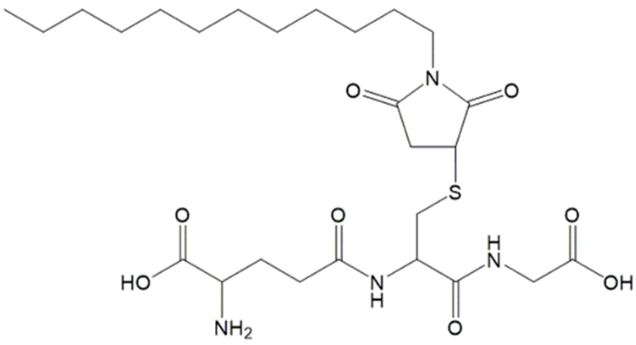

Figure S1. Structure of GSH-NLM.

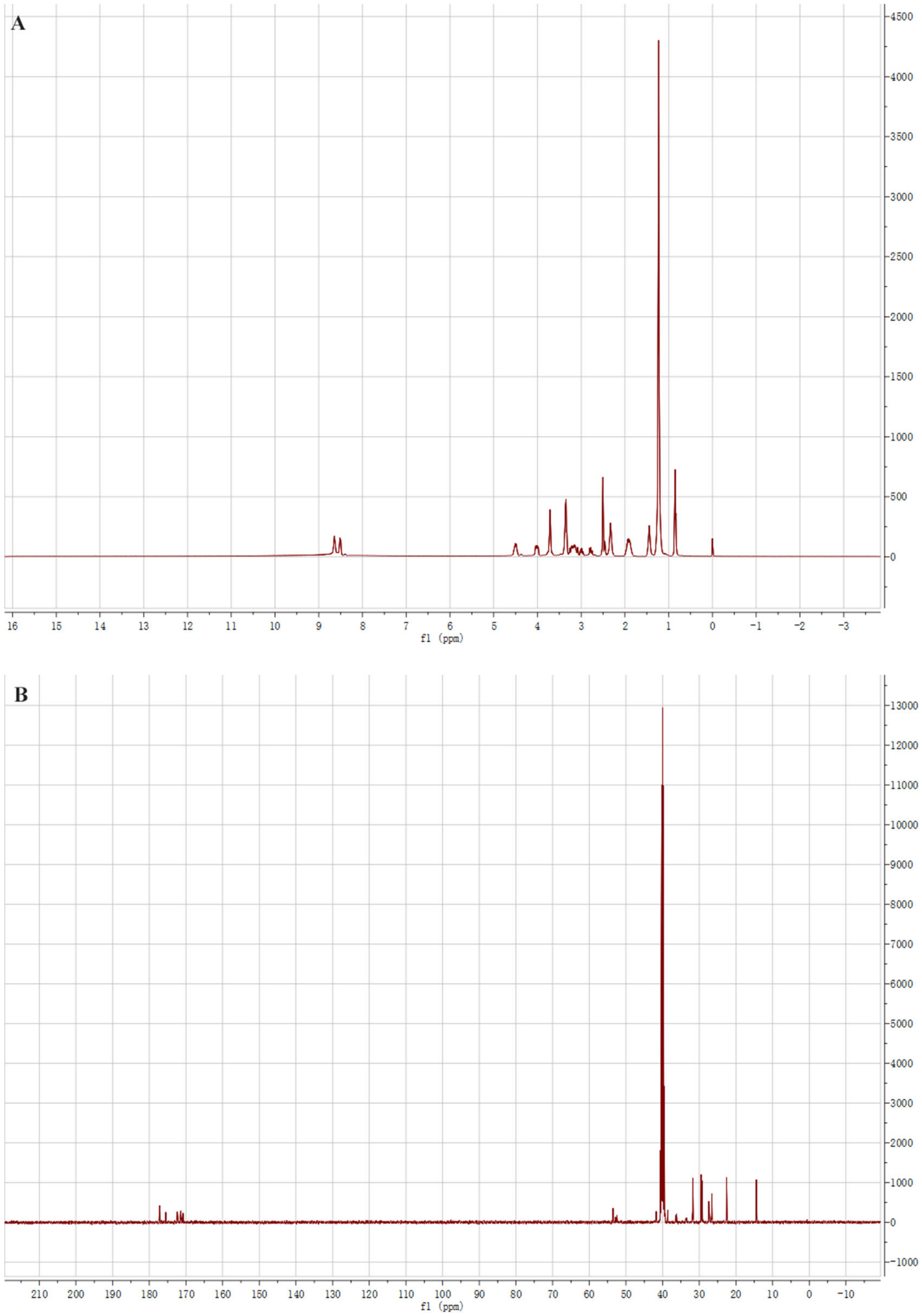

Figure S2. (A) ^{1}H-NMR and (B) ^{13}C-NMR spectra of GSH-NLM.

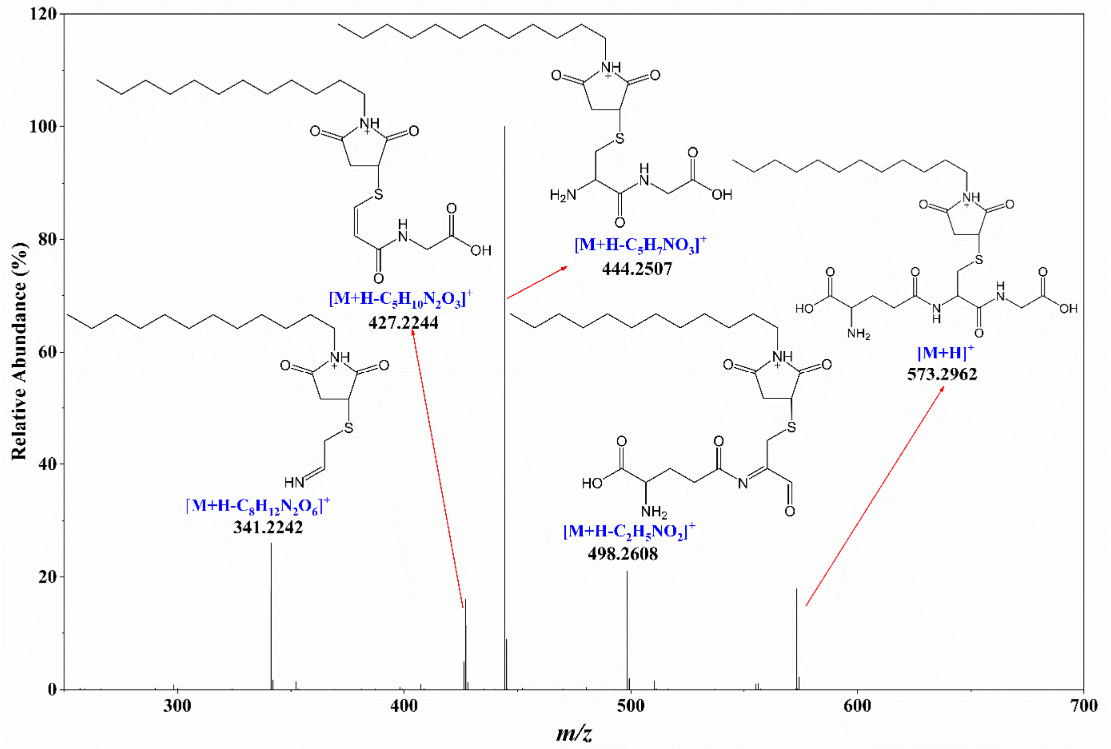

Figure S3. Product ion spectrum of protonated GSH-NLM at *m/z* 573.2962.

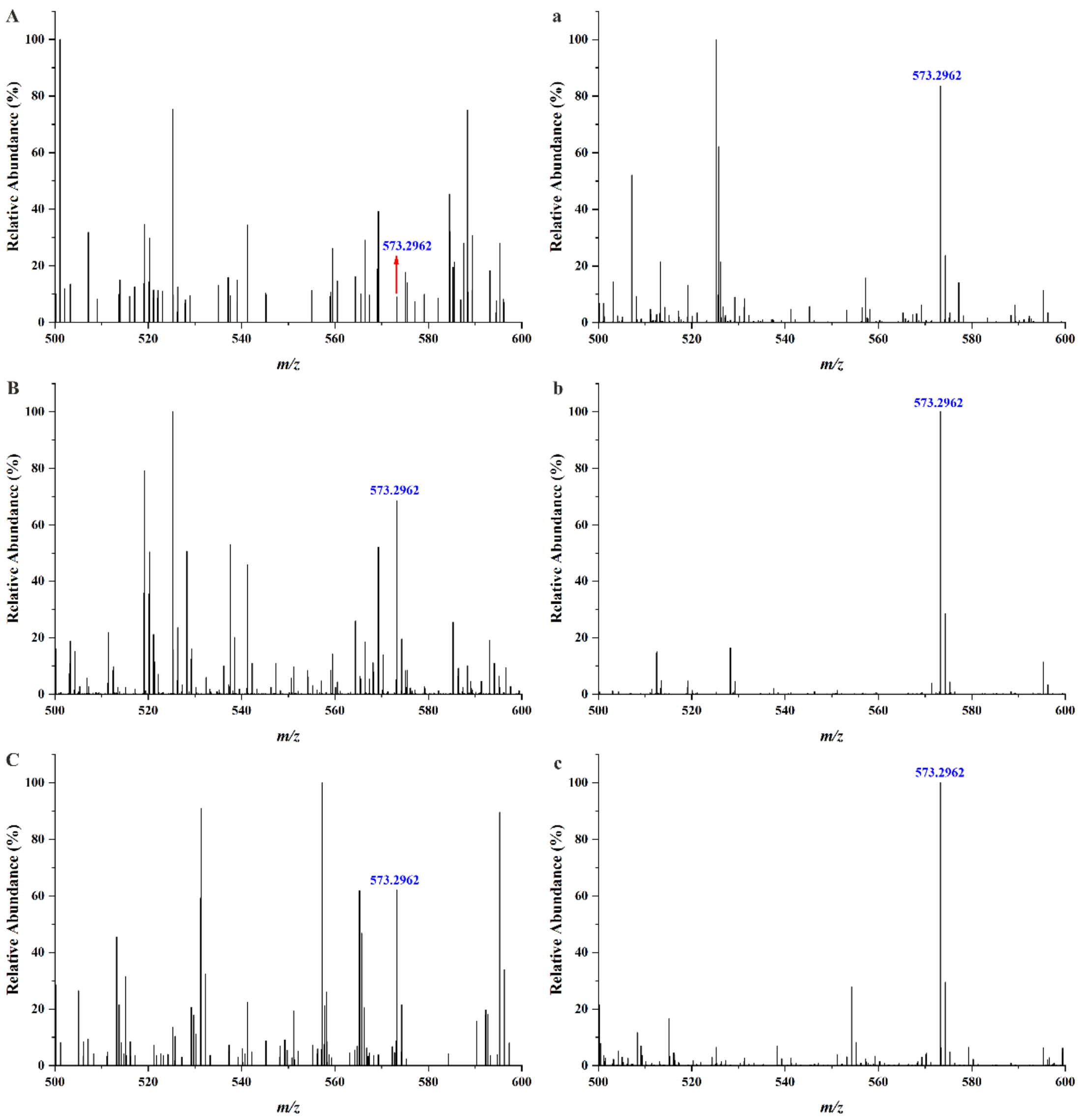

Figure S4. Mass spectra of artificial biofluids spiked with GSH-NLM, exhibited within the range of *m/z* 500–600: saliva treated (A) without and (a) with THF; urine treated (B) without and (b) with THF; plasma treated (C) without and (c) with THF.

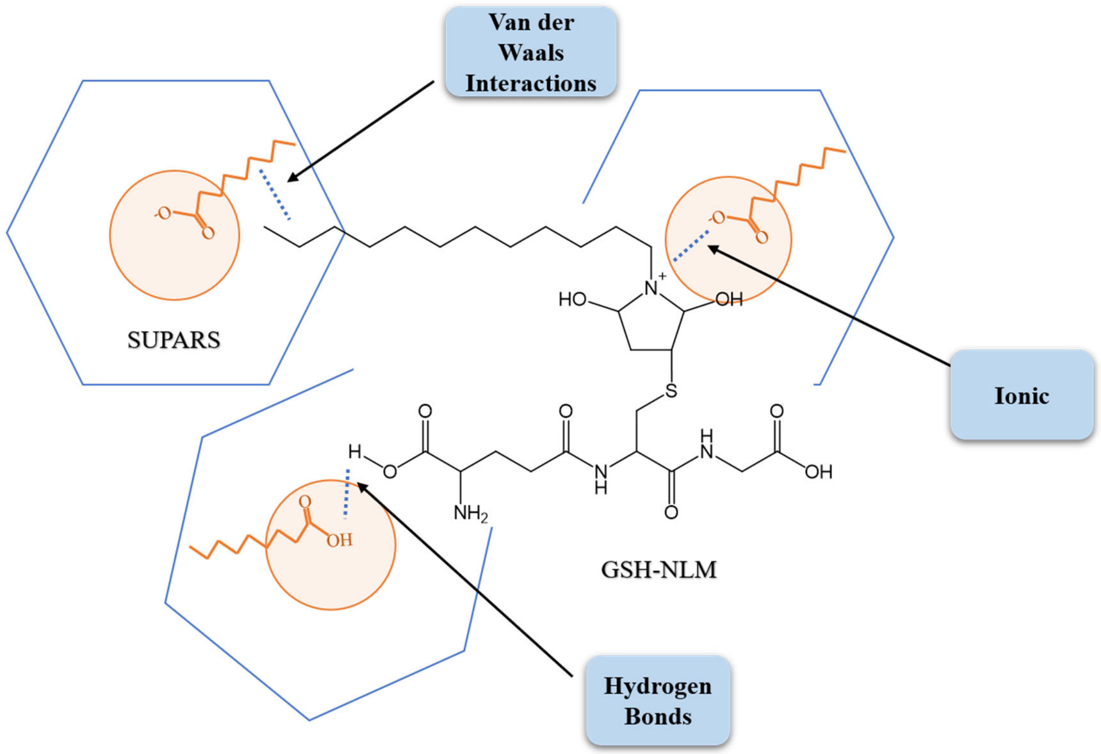

Figure S5. Proposed interactions between GSH-NLM and SUPRAS during the DLLME process.

References

Rahman, Kode and Biswas (2006). Assay for quantitative determination of glutathione and glutathione disulfide levels using enzymatic recycling method. Nat. Protoc. 1(6), 3159-3165. doi:10.1038/nprot.2006.378

Cai, Li, Ge, Zhang, Hu, Li and Qu (2015). A rapid fluorescence "switch-on" assay for glutathione detection by using carbon dots–MnO_2 nanocomposites. *Biosens. Bioelectron.* 72, 31-36. doi:10.1016/j.bios.2015.04.077

Yuan, Zeng, Xu, Liu, Ma, Zhang and Fan (2013). Electrochemical modification of graphene oxide bearing different types of oxygen functional species for the electro-catalytic oxidation of reduced glutathione. Sensors Actuators B: Chem. 184, 15-20. doi:10.1016/j.snb.2013.04.055

Zhu, Wu, Wang, Xu and Xie (2020). Facile and sensitive measurement of GSH/GSSG in cells by surface-enhanced Raman spectroscopy. *Talanta* 224, 121852. doi: 10.1016/j.talanta.2020.121852
